# Chemical Sensing Using Fiber Cavity Ring-Down Spectroscopy

**DOI:** 10.3390/s100301716

**Published:** 2010-03-02

**Authors:** Helen Waechter, Jessica Litman, Adrienne H. Cheung, Jack A. Barnes, Hans-Peter Loock

**Affiliations:** Queen’s University, Department of Chemistry, Kingston, ON, K7L 3N6, Canada; E-Mails: wachterh@chem.queensu.ca (H.W.); Jessica.Litman@chem.queensu.ca (J.L.); 6ac18@queensu.ca (A.H.C.); jbarnes@chem.queensu.ca (J.A.B.)

**Keywords:** cavity ring-down (CRD), absorption, refractive index, fiber-loop, fiber cavity, microfluidics, capillary electrophoresis, phase-shift, long-period grating (LPG), fiber Bragg grating (FBG), PACS 07.60.Vg, 42.62.Fi, 42.81.Pa, 42.60.Da, 82.80.Dx, 78.20.Ci

## Abstract

Waveguide-based cavity ring-down spectroscopy (CRD) can be used for quantitative measurements of chemical concentrations in small amounts of liquid, in gases or in films. The change in ring-down time can be correlated to analyte concentration when using fiber optic sensing elements that change their attenuation in dependence of either sample absorption or refractive index. Two types of fiber cavities, *i.e.*, fiber loops and fiber strands containing reflective elements, are distinguished. Both types of cavities were coupled to a variety of chemical sensor elements, which are discussed and compared.

## 1. Introduction

Cavity ring-down (CRD) spectroscopy and related cavity-enhanced absorption spectroscopic methods have garnered a large following in the past decade [1–3]. A typical CRD setup involves an optical cavity made from two [4] or three [5,6] highly reflective mirrors. Light is coupled into the cavity through one of the mirrors and light leaking out of the cavity is detected. When a cavity resonance is excited, power builds up in the cavity and then decays exponentially as the light source is switched off. The decay time (ring-down time, *τ*) is a direct measure of the optical loss in the cavity. In cavity ring-down spectrometers the optical loss is changed by filling the cavity, at least in part, with an absorber, which can then be quantified.

Most CRD spectrometers are designed for very sensitive gas absorption measurements and have volumes of tens to hundreds of milliliters. Measurements on liquid samples require either filling the entire cavity with liquid [7,8] or placing the sample into a part of the optical cavity, e.g., contained by a cuvette [9–12], or in a free flowing liquid sheet [13,14]. In the latter experiments the cavity needs to be carefully aligned to reduce the losses due to the liquid, the interfaces and the sample containers. For very small liquid samples that are relevant to e.g., high-performance liquid chromatography (HPLC) and capillary electrophoresis, there is no benefit in using large cavity volumes, and cavities have been built with reduced distance between the mirrors such that the cavity volume is comparable to the detection volume [15].

An alternative approach to mirror-based CRD detection for condensed phase absorption and refractive index measurements was developed by our group and others since 2002. In fiber-cavity ring-down spectroscopy the optical cavity consists of a strand of single-mode or multi-mode fiber optic cable. The light is trapped in the cavity by either using “mirrors” (*i.e.*, reflective coatings [16] or fiber Bragg gratings [17]) at the ends of a linear fiber strand, or by connecting the ends to form a loop, *i.e.*, a ring cavity [3,18,19]. Similar to “free-space” CRD, light can be trapped in the fiber and its ring-down time decreases with increasing optical loss in the waveguide cavity. Fiber Bragg grating (FBG) cavities have a very narrow bandwidth of only about 2–5 nm; therefore they are limited in their use for spectroscopic measurements. However, they are easy to align and are well suited for mechanical measurements. Conversely, fiber loops are inherently broadband cavities and have a low roundtrip loss over the entire transmission range of the fiber material - for silica fibers from approximately 1.7 μm to 250 nm. Such a fiber loop cavity can be designed to hold a variety of sensor heads, such as very small absorption cells [20], long-period gratings for refractive index sensing [21], evanescent field blocks [17], tapers for absorption [22,23] or strain [24] sensing, and others.

Conventional mirror-based CRD spectroscopy achieves remarkable detection limits with minimum detectable absorption losses α_min_ = 10^−11^ cm^−1^ in some cases, whereas fiber-cavity ring-down spectroscopy is by many orders of magnitude less sensitive (α_min_ = 0.1 cm^−1^ in the experiments described in section 2.2). We believe that the technique is only advantageous when the sensor head consists of a fiber optic element (described in sections 2.1, 2.3 and 2.4) or when a sample with dimensions comparable to that of the fiber is interrogated, such as the liquid samples in section 2.2. In fiber-CRDS nanoliters or even picoliters of sample are sufficient [20], whereas even the smallest mirror cavities require several microliters of sample [8].

For non-fluorescing liquid samples single-pass absorption spectroscopy is the standard detection and quantification method. Commercial systems offer microliter detection volumes and by using two balanced detectors high sensitivities can be achieved. For example, a modern UV-VIS detector (Agilent 1200 Series multiple wavelength detector) has a short term noise of 0.8 × 10^−5^ absorption units, corresponding to α_min_ = εC = 2.4 × 10^−5^ cm^−1^ (3σ) when combined with a standard flow cell (1 cm pathlength, 13 μL detection volume). Again, the detection volume in such a detector is three to four orders of magnitude larger compared to fiber-CRDS and is comparable to the entire sample volume used in fiber-based systems. To take advantage of the small detection volumes in fiber-CRD the method is best coupled to separation systems in which the sample volume is restricted to microliters and the detection volume cannot exceed a few tens of nanoliters, such as capillary electrophoresis and microfluidic devices (see section 2.2).

In this review we will first present a brief overview of the theoretical foundations and then discuss different designs of sensor elements for fiber-based CRD devices and their applications.

### 1.1. Theory

Cavity ring-down spectroscopy is based on determining the optical losses of a high-finesse cavity by monitoring the lifetime of a photon trapped in the cavity. The photon lifetime is related to absorption and scattering of the light in the cavity but it is independent of intensity fluctuations or detection efficiency. The photon lifetime or ring-down time *τ* is given by the round trip time of the cavity *t_RT_* = *nL/c_0_* (*L*: cavity length, *n*: effective refractive index of the cavity medium, *c_0_*: speed of light in vacuum) and the transmission per round trip *T_rt_*.

(1) $$\tau =\frac{t_{\mathrm{RT}}}{-\text{ln}\;\left(T_{\mathrm{rt}}\right)}$$

The transmission per round trip *T_rt_* is governed by the losses arising from the cavity itself, such as losses at the mirrors, and by sample absorption within the cavity.

In fiber-based CRD setups an optical cavity is made of an optical fiber containing a sensing element that allows the light to interact with the sample. This can be done, for example, by placing the sample in a gap in the fiber, by evanescent wave absorption, or by measuring the losses in a fiber grating that depend on the refractive index of the surrounding material. The ring-down times in fiber cavities are determined by the losses in the sensor element and the absorption coefficient of the fiber *a_f_*.

(2) $$\tau =\frac{\mathrm{nL}}{c_{0}\left(-\text{ln}\;T_{\mathrm{sensor}}+\alpha_{f}L\right)}$$

The transmission *T_sensor_* across the sensor element contains contributions from the “undesired” insertion losses of the sensor element and the “desired” loss from interactions with the sample. For example, if the sensor head contains a small absorption cell, the loss term is given by

(3) $$-\text{ln}\;T_{\mathrm{sensor}}=-\text{ln}\;T_{\mathrm{gap}}+\tilde{\varepsilon }\mathrm{Cd}$$

Here, the term −ln *T_gap_* describes coupling losses across the gap, and *ε̃Cd* is the Lambert-Beer term that depends on the interaction length through the sample cell *d*, the concentration *C*, and the extinction coefficient *ε̃_s_* given with respect to base *e*, which is related to the decadic extinction coefficient *ε_s_* by *ε̃_s_* = *ε_s_* ln(10). The relationship between the ring-down time, *τ* and the sample concentration, *C,* is then obtained by applying equation (3) to equation (2).

In free-space cavities the ring-down event is usually well described with a single exponentially decaying function. Different cavity modes have different ring-down times, but with careful alignment only the fundamental mode may be excited. In fiber cavities, on the other hand, there is light traveling in the core, cladding and sometimes in the buffer jacket leading to biexponential or triexponential decays. Longitudinal cavity mode structure may be easily resolved for short (<1 m) fiber cavities made of single mode fibers [17], but is not apparent for multimode fibers where hundreds or thousands of core modes are simultaneously excited. The intensity of a multiexponential decay with ring-down times *τ_i_* and amplitudes *a_i_* can be expressed as:

(4) $$I(t)=\sum_{i}^{N}a_{i}\text{exp}\left(-t/\tau_{i}\right)$$

Each ring-down time *τ_i_* is related to the sample concentration *C,* as in the single exponential decay case, but the insertion losses of the sensor element and the absorption losses due to the fiber are different for each decay time *τ_i_*.

### 1.2. Phase-Shift Cavity Ring-Down Spectroscopy

**Figure 1.**
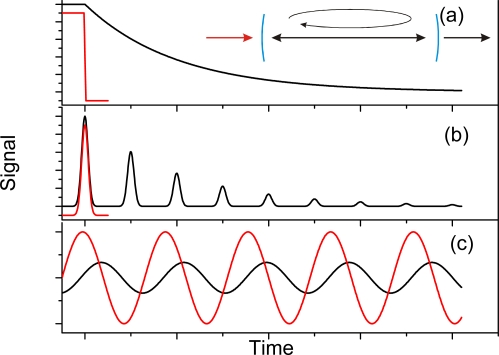
Different implementations of cavity ring-down measurements **(a)** In time-resolved continuous wave CRD the light entering the cavity (see inset) is switched quickly (red line), and the intensity decay in the cavity is monitored from the cavity build-up time or the ring-down time (black). **(b)** If the injected pulse (red) is short compared to the round-trip time, *t_rt_*, the ring-down time is obtained from the exponential decay constant of the amplitudes of the light pulses that are leaked from the cavity with each round trip (black). **(c)** In phase-shift cavity ring-down the light intensity of a cw-source is modulated sinusoidally (red). The cavity introduces a phase-shift (black) that is related to the losses in the cavity and therefore to the ring-down time.

Time-resolved CRD spectroscopy may not be ideal when measurements need to be conducted with a high sampling rate; even for a high repetition rate laser (10 kHz) and a long ring-down trace of 10 μs, the duty cycle is only 10%. An alternative to time-dependent measurements of the ring-down time is the frequency equivalent, *i.e.*, instead of determining the decay constant from the time response of the cavity to a pulse, one can determine the phase shift that a sinusoidally modulated waveform experiences when traversing the cavity (see Figure 1). Phase-shift measurements were initially used in 1980 by Herbelin *et al*. to characterize optical cavities [25] and were later implemented for optical absorption measurements by Engeln *et al*. [26]. For single-exponential decays the relationship between the ring-down time *τ*, the modulation frequency *ω* and the phase-shift *ϕ* is given by [26,27]:

(5) tan (ϕ)=−ωτ

For a multiexponential decay the relationship between the ring-down times *τ_i_*, the amplitudes *a_i_* and the phase shift *ϕ* is [28]

(6) $$\text{tan}\phi =\frac{-\sum_{i}^{N}\frac{{\omega a}_{i}\tau_{i}^{2}}{\omega^{2}\tau_{i}^{2}+1}}{\sum_{i}^{N}\frac{a_{i}\tau_{i}}{\omega^{2}\tau_{i}^{2}+1}}$$

The phase-shift method is particularly suitable for fast measurements in liquid flow samples, since the duty cycle of phase-shift CRD is unity and the data acquisition rate is fundamentally limited only by the ring-down time. Furthermore, the phase-shift method is compatible with very short ring-down times, which may be readily measured using high modulation frequencies. Sinusoidal modulation at 10–100 MHz frequencies allow accurate measurements of (sub-)nanosecond ring-down times and is easily implemented for a variety of light sources including cw-lasers and LEDs. The largest disadvantage of phase-shift CRD is the inability to discriminate multiple exponential decays from a single measurement. Ring-down times for multiexponential decays must therefore be extracted from phase-shift measurements at more than one modulation frequency if needed [28]. On the other hand, for many analytical measurements calibrating the system with known concentrations at a fixed modulation frequency may be sufficient.

### 1.3. Properties of Fiber Cavities

**Figure 2.**
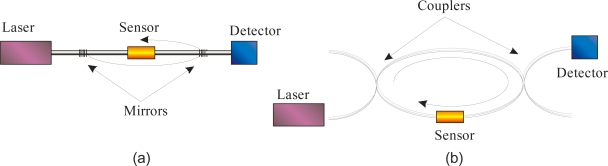
**(a)** Setup of a linear fiber cavity. Two mirror elements, such as fiber Bragg gratings, are incorporated into the fiber to form an optical cavity. The light is injected through one of the mirrors and is detected behind the second mirror. **(b)** Setup of a fiber loop cavity. The fiber is bent into a loop to form a ring cavity. Fiber couplers with high splitting ratios may be used to inject light into the loop and to direct a small fraction of the light to the detector.

For the purpose of this review we distinguish between two types of fiber cavities: (1) the linear fiber cavity where light is confined by fiber optic “mirrors” such as reflective coatings or fiber Bragg gratings (FBGs) and (2) a loop of waveguide material (see Figure 2).

As an example for the linear fiber cavity, von Lerber *et al.* [16] applied dielectric coatings on the fiber ends that acted as mirrors and achieved remarkably low roundtrip losses of 0.42%. At about the same time Gupta *et al*. [17] used fiber Bragg gratings (FBG) as cavity mirrors. FBGs have a strongly wavelength dependent attenuation (up to 40 dB, corresponding to a reflectivity of 99.99%) and most of the light is reflected into the single counter-propagating core mode. With a 10 m long hydrogen-loaded single-mode fiber Gupta *et al*. achieved 2.3 % roundtrip loss. Nowadays, most linear fiber cavities use FBGs as reflectors since they are commercially available and easy to implement. Linear FBG cavities have other advantages. First, very short cavities of a few millimeters can be built. By comparison, the minimum cavity length for fiber loops, described below, is limited by macrobending losses to approximately 30 cm. Second, it is straightforward to couple light into and out of these cavities, since the light source and detector can be placed in-line with the FBG cavity. Third, the FBG itself can be used as sensing element since it is sensitive to thermal expansion and pressure [29]. On the other hand, FBGs reflect light only within a very limited wavelength range of a few nanometers, and they are commercially available only at telecom wavelengths. This severely limits the range of wavelengths for e.g., absorption measurements and poses problems with light sources that have an ASE background. The broadband ASE contribution may only be 10^−6^ of the light source intensity, but since it falls largely out of the FBG reflection range its contribution to the signal may be as large as the cavity signal that is nominally also attenuated to 10^−6^ of the incoming light, if the FBGs are 99.9% reflective (30 dB transmission loss) [28]. Finally, commercially available FBGs are usually designed for single-mode fibers, and multimode fibers or other specialty fibers cannot be used with commercial FBGs.

A cavity can also be obtained by forming a loop of waveguide, *i.e.*, a ring cavity. Similar to the FBG cavity, the fiber loop cavity is simple, rugged and inexpensive. To decrease the roundtrip loss, the fiber ends may be connected using a fusion splicer or a commercial mechanical connector [19], but it is also possible to leave a gap in the loop where samples can be injected. Fiber couplers with high split ratios (e.g., 99:1) can be spliced into the loop to couple light into the fiber cavity. Alternatively, focusing a laser beam on the loop fiber also allows coupling of a small fraction of light (about 1:10^8^) into the loop [18]. To measure the light intensity in the loop a photodetector is either placed close to the loop to detect scattered light from the fiber, or a (second) fiber coupler is spliced into the loop to direct a small fraction of the light to the detector.

In contrast to the FBG cavity, the fiber loop is a “broad band” cavity. The transmission of the fiber loop is only limited by the transmission of the fiber-optic material and the components. A multimode silica fiber cavity has a usable transmission from approximately 250 nm to 1,700 nm, depending on the OH content in the silica. At telecom wavelengths (1.3–1.5 μm) a large variety of fibers exist with absorption losses as low as 5 × 10^−4^ dB/m; at UV-wavelengths the losses are considerably higher even for special UV-fibers (e.g., 0.05 dB/m at 400 nm, 0.3 dB/m at 250 nm), but ring-down experiments are still possible. More so, specialty fibers are available for the mid-infrared region that have absorption losses of less than 0.5–0.2 dB/m and make experiments at wavelength up to 9 μm possible. To date no research group has taken full advantage of the broadband properties of the fiber loop, but it may be expected that multi-wavelength detection will be attempted soon.

The main disadvantage of fiber loop cavities compared to linear cavities lies in the trade-off between high coupling efficiency and high roundtrip loss. The losses in the fiber loop should be kept as low as possible for sensitive ring-down time measurements, but couplers and splices are nevertheless needed to inject sufficient light into the cavity. These components introduce additional losses (fusion splices about 0.02 dB, mechanical splices about 0.23 dB), and in our experience even fiber couplers with a high split ratio of 99:1 may add insertion losses of 2–4% to the roundtrip loss.

## 2. An Overview of Types of Sensors and Applications

**Figure 3.**
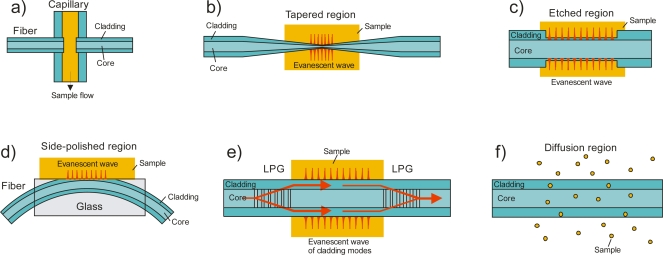
Different types of sensor elements used in fiber-cavity ring-down spectroscopy. **(a)** A gap in the fiber allows for direct absorption of all propagated light through the sample. **(b)** In a tapered fiber the core mode is converted into a cladding mode and the evanescent field extends outside the fiber. **(c)** By etching the cladding the evanescent field can also be extended into the sample. **(d)** In a field access block the cladding thickness is reduced by side-polishing the fiber and the evanescent field then reaches the surrounding material. **(e)** A pair of identical long-period gratings (LPGs) couples light from the core mode into a single cladding mode and then back into the core. Between the two LPGs the light travels in the cladding mode and its large evanescent wave interacts strongly with the surrounding sample. **(f)** Gases of small molecules such as H_2_ can diffuse into the fiber and cause absorption that can be monitored.

For both the linear cavity and the fiber loop cavity the circulating light can interact with a chemical sample (see Figure 3). For example, the sample can be placed in a gap in the fiber cavity and the ring-down time changes due to optical absorption, scattering or dispersion. Absorption and scattering may also be measured by exposing the immediate environment of the fiber to the evanescent field of the propagating mode. The evanescent field of the core mode(s) is usually contained in the cladding to achieve low propagation loss, and for absorption measurements it will have to be extended to penetrate the environment of the fiber or the fiber cladding needs to be removed.

Other sensor heads consist of fiber optic components that react to the refractive index of the environment [21]. Finally, fiber optic components inserted into the cavity may be sensitive to temperature [30], pressure [31,32], bending losses [19,33] and strain [34,35] exerted on the fiber. A recent review described mechanical and thermal fiber sensors [36]; here we focus on fiber optic cavities that contain elements suited for chemical detection and sensing.

### 2.1. Direct Absorption Measurements

In 1982 the diffusion of hydrogen gas into silica waveguides caused unexpected attenuation in the first field-installed optical fibers [37]. Hydrogen is produced by chemical reactions of the constituents of the cable material and then diffuses into the fiber. Vogler *et al*. [38] examined the diffusion rate of hydrogen into optical fibers using fiber cavity ring-down spectroscopy. They used a single-mode silica fiber cavity (*L* = 10 m) with two highly reflective dielectric mirrors coated onto the fiber ends. Pulsed light from an external cavity diode laser (1,520–1,600 nm) was injected into the cavity through one of the mirrors and the decay of the transmitted light intensity was monitored by a photodiode. Ring-down times of typically 10 μs were observed corresponding to a loss of 0.021 dB per round trip. An effective interaction length of 2*L* τ/*t_RT_* = 2070 m is calculated with this ring-down time and the roundtrip time *t_RT_* = 96 ns. The fiber was coiled on a mandrel and was saturated with the gas by exposing it to pure hydrogen at 1 bar for two weeks. The fiber was then placed in a hydrogen-free environment at 30 °C and H_2_ slowly diffused out of the fiber. By monitoring the ring-down time the optical loss due to the hydrogen absorption and the diffusion constant (3.02 ± 0.07 × 10^−15^ m^2^/s) were determined.

### 2.2. Liquid Absorption Using a Microcell in the Fiber Cavity

Standard fiber-optic cables have a core diameter between about 8–400 μm, *i.e.*, their dimensions are comparable to those of flow channels in micro-separation systems, such as capillary electrophoresis (CE), micro-liquid chromatography or microfluidic devices (“lab-on-a-chip”, or micro-total analysis systems, μ-TAS). The gap that is formed between the fiber ends when making a fiber loop cavity is a natural place to insert a small liquid sample and, consequently, waveguide-CRD or fiber loop ring-down spectroscopy has been used for absorption detection coupled to a variety of microseparation systems [3,18–20,39].

The dimensions of the sample gap should be similar to those of the flow channel to prevent pressure build-up or turbulences. Also, to achieve a good transmission across the gap, the diameter of the waveguide core needs to be equal to or larger than the width of the sample gap. Capillaries used for CE have inner diameters ranging from 50 to 100 μm, whereas commercial microfluidic devices have flow channel dimensions of typically 30 × 50 μm. Therefore, for these separation systems regular multimode fibers with core diameters of 30 to 100 μm can be used as cavity medium. Emerging nanofluidic separation system use much smaller channels with cross sectional dimensions <1 μm, whereas conventional HPLC uses much larger flow channels with dimensions of millimeters. In the latter case conventional CRD with very small mirrors demonstrated remarkable sensitivity of about α_min_ = 1.5 × 10^−4^ cm^−1^ [15,40]. Recently, micro-HPLC (flow channel diameters: 50 to 500 μm) has become increasingly popular, since it uses smaller sample volumes, allows higher pressures, and consumes less solvent compared to regular HPLC. Waveguide CRD is quite amenable to absorption detection in the sub-microliter detection volumes found in micro-HPLC.

The principle of intersecting a fiber loop with a sample flow appears simple (see Figure 3a), but the design of such an interface is quite challenging. The fiber ends need to be aligned carefully to achieve a good transmission through the sample gap and the flow channel has to be connected without causing a pressure build-up or a large dead volume. Especially the lateral alignment of the fiber ends is critical as a misalignment can cause large losses. For example, lateral misalignment by 10% (displacement/fiber diameter) leads to additional transmission losses of >10% but requires a precision of 5 μm for a multimode fiber with a core diameter of 50 μm or a precision of <1 μm for a single mode fiber [41,42].

In one of our first fiber loop CRD experiments [18,39] the fiber ends of a multimode fiber loop (length of 65 meters, core diameter 400 μm, cladding diameter 440 μm) were joined by a commercial four-way microcross. The two fiber ends were inserted through opposing holes and two capillaries (inner diameter 100 μm) for the sample flow were inserted through the other two holes. The detection volume is defined by the distance between the fiber ends and the core diameter. The gap between the fiber ends was 31 μm, which created a detection volume of 4 nL. Samples of the laser dye 1,1′-diethyl-4,4′-dicarbocyanine iodide (DDCI) in dimethyl sulfoxide (DMSO) were measured with phase-shift CRDS at different concentrations at a wavelength of 810 nm. A detection limit of *C_min_* = 6 μM DDCI in DMSO was obtained, which corresponds to an absorbance of *ε* · *C_min_* = 2.0 cm^−1^. Since the upper concentration limit was 5.5 mM, we determined a dynamic range of 3 orders of magnitude for this system.

Later our group used a similar system as an online detector for CE of biomolecules [43]. The interface to the liquid was fabricated by drilling holes through a polymethyl methacrylate (PMMA) block, where the capillary and the two ends of the fiber strand were inserted and affixed using epoxy glue. The fibers were aligned to the capillary walls at a distance of 30 μm. To increase the transmission through this sample gap, a microlens with a radius of 76 μm was made at one of the fiber ends by melting the end in the electric arc of a fusion splicer. A fiber coupler with a split ratio of 99:1 was used to couple 810 nm light into the loop. Measurements were performed using capillary electrophoresis to separate dye-labeled human serum albumin (HSA) from the unbound NIR dye (ADS805WS). The excess free dye and the dye/protein complex were resolved using a run buffer of 10 mM boric acid with pH 10.0, the labeling coefficient was determined to be ∼6. The detection limit was 1.6 cm^−1^, corresponding to 1.67 μM of the labeled HSA.

The above experiments all operated at near-infrared wavelengths, which limits the variety of substances that can be detected. More recently, the fiber loop ring-down method was adapted to shorter wavelengths. For example, Andachi *et al*. [44] used a picosecond pulsed diode laser at 660 nm with a fiber loop cavity. Two erbium doped fiber amplifiers were used in series to obtain stable and intense pulses of 40 ps duration by gain-switching. The fiber loop was made from 4.69 m of multimode fiber with a 50 μm core diameter. Two fiber couplers with a 99:1 split ratio were spliced into the loop, one for injecting laser light into the loop and the second one for directing a fraction of the light to a photomultiplier tube. The fiber loop contained a microcell (gap width: 100 μm, detection volume: 200 pL) to introduce a liquid sample between the fiber ends. A biexponential decay was observed with ring-down times of 399 ns (0.247 dB loss per round trip) and 118 ns (0.834 dB loss) corresponding to the decay of core and cladding modes, respectively. Measurements of methylene blue in an aqueous solution were performed at a wavelength of 660 nm. The detection limit was determined to be 50 μM, which corresponds to an absorption loss of 6 cm^−1^.

**Figure 4.**
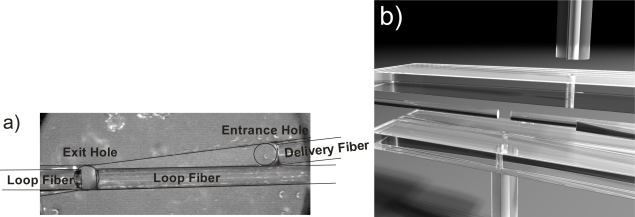
A custom interface is used to direct liquid between the fiber ends and to couple light from the light source into the loop. The delivery fiber irradiates the fiber loop ends so that some of the light enters the loop. The sample is injected through a hole in the top plate in front of the delivery fiber, flows along the groove to the gap between the fiber ends and exits through a hole in the bottom plate [20]. **(a)** Top view. **(b)** Computer generated schematic image.

Our latest system [20] operates at 405 nm since many biomolecules (e.g., proteins) have strong absorption features in this region. The optical attenuation of UV waveguides is orders of magnitude higher compared to near-IR waveguides. We designed a system that consisted of a comparably short fiber loop (10 m length), a photomultiplier tube attached to a bend in the fiber, and a fiber-coupled diode laser. Unfortunately, fiber optic couplers for multimode UV-waveguides are not commercially available. Therefore, we devised an interface that let us introduce the sample liquid and the light into the loop at the same time. In the interface the two fiber loop ends were placed 190 μm apart in a groove that was cut into PMMA polymer. Another fiber coming from the laser was affixed into a second groove that had been cut at a narrow angle to this sample gap. Here, it irradiated one of the fiber ends to couple light into the loop (see Figure 4). The sample entered the interface through a hole in the top plate in front of the delivery fiber, flowed along the groove and between the gap in the fiber loop and then exited the interface through a hole below the sample gap. The detection volume was given by the distance between the fiber ends and the core diameter and had a size of 6.0 nL. This interface avoids introducing additional losses due to a fiber coupler and can be used with any type of fibers, including mid-infrared fibers or even photonic crystal fibers.

**Figure 5.**
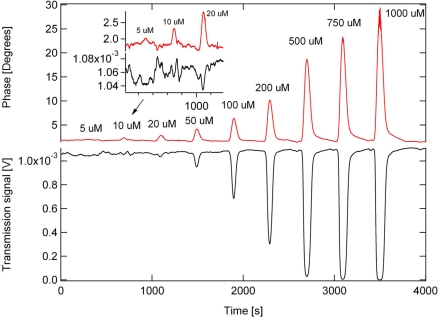
Measurements of tartrazine at 405 nm with phase-shift CRD (red curve, top) and single pass absorption (black, bottom). Samples (5 μL) of different concentrations were injected into a flow of water (flow rate: 10 μL/min) with a sample injector (Rheodyne) [20].

Phase-shift cavity ring-down detection was used because of the higher data acquisition rate and because short ring-down times could be compensated by increasing the modulation frequencies (modulation frequency: 1 MHz here, τ = 140 ns, effective absorption path: 550 μm). Measurements of tartrazine (yellow food dye), myoglobin and a pharmaceutical component provided by *Eli Lilly, Canada*, were performed at low micromolar concentrations (see Figure 5).

A detection limit of 5 μM tartrazine or 1 μM myoglobin is achieved; this corresponds to an extinction of 0.11 cm^−1^ or 30 femtomoles of tartrazine in the detection volume. At the same time transmission spectroscopy was performed on the sample flowing along the groove (path length: 3 mm). Despite the 15-fold larger absorption path (and sample volume!) for the transmission measurement, the ring-down measurement has a four-fold higher sensitivity and a larger dynamic range. The direct transmission measurement is limited by laser power fluctuation to which CDRS is insensitive. For a more accurate transmission measurement, a second detector would be needed to normalize the laser power.

### 2.3. Evanescent Field Absorption

Instead of leaving a gap in the fiber cavity, which introduces large losses on its own, it is possible to use the evanescent wave of the circulating light for concentration measurements. The evanescent wave can interact with the surrounding medium leading to cavity losses, either by attenuation (absorption and scattering) of the evanescent field, or by dissipation of the energy, if the refractive index of the samples approaches that of the cladding. By measuring the ring-down time the cavity losses due to the evanescent wave interaction can be determined. Usually the evanescent field of the core mode(s) is contained entirely inside the cladding; thus for a measurable interaction with the sample it needs to be extended into the surrounding medium. This can be achieved by tapering, etching or side-polishing the fiber (see Figure 3). An effective absorption path can be obtained from the evanescent field depth of all guided modes [45]. For a single-mode fiber, the evanescent field of the core mode can be obtained directly from its mode field diameter [3].

Sigrist’s group [16] demonstrated the use of fiber CRD spectroscopy in combination with evanescent field absorption for chemical sensing applications. As the sensing element an etched single mode fiber was used in a linear cavity with a length of 0.576 meters. High reflectivity dielectric coatings (R > 99.9%) on both fiber ends acted as cavity mirrors. The sensor was prepared by stripping the coating from the fiber and etching in buffered hydrofluoric acid solution (caution: extremely dangerous). The evanescent field experiences absorption due to the water present in the etch solution. With decreasing fiber diameter, the evanescent tail expands into the solution, the absorption increases, and the ring-down time decreases. Before etching the fiber diameter was 125 μm and the ring-down time was 755 ns corresponding to a round trip loss of 0.016 dB. When the fiber was etched to a diameter of 63 μm the ring-down time was reduced to 550 ns (0.022 dB). A detection limit of 4.68 × 10^−4^ dB loss was achieved.

Field access blocks have also been used to extend the evanescent field into the sample. Here, the fiber is side-polished to remove a part of the cladding. Gupta *et al*. [17] designed a single mode fiber cavity using fiber Bragg gratings (FBG) as mirrors and a 6.9 cm long evanescent-wave access block for chemical sensing. The block was immersed in a glycerin/water mixture so that the external refractive index could be varied from 1.4746 (pure glycerin) to 1.330 (pure water). From the increase of cavity losses with increasing refractive index a cavity gain factor of about 100 was determined.

Tapered fibers are a third method to extend the evanescent wave into the surrounding sample. Tarsa *et al*. [22] developed a fiber loop ring-down system with a tapered fiber region. The resonator consisted of a 2.2 km long single mode fiber, two fiber couplers with 99:1 split ratio and a tapered section. The taper was 28.0 mm long, has a waist radius of 10 μm, and was slightly bent to enhance the evanescent field. The small waist allowed complete conversion of the core mode to an evanescent cladding mode. For absorption measurements the taper was immersed in 1-octyne with an absorption line at 1532.5 nm. By scanning across this absorption line, the concentration was determined with a detection limit of 1.05% 1-octyne, corresponding to an absorption of 260 cm^−1^. The same setup was also used for biological cell detection [23]. Cells have a nucleus with a high optical density that leads to efficient scattering. The scattering loss varies with cell size and shape and the amount of high refractive index organelles. In order to localize the cells on the tapered region, the fiber taper was coated with a polypeptide (poly-D-lysine) that is optically transparent and has a strong binding affinity for cellular membrane proteins. Tarsa *et al*. performed measurements on mammalian cancer cells since they are of considerable interest, are relatively large (10 μm) and have high melanin content. By adding trypsin the adhered cell can be detached from the coated fiber. After the cell was attached to the coating the surrounding solution was removed to increase the difference in refractive index. The ring-down time changed linearly for 0 to 150 cells attached to the fiber. The rate of change in the ring-down time was 0.23 μs/cell, thus allowing single-cell detection.

Another method uses identical pairs of long-period gratings (LPG) to extend the evanescent field by converting the core mode into a specific low-order cladding mode - depending on the period of the grating and the wavelength of the light [46]—and then recouple into the core mode. The device would form a Mach-Zehnder interferometer, if the light was split approximately evenly between the core and the single cladding mode [47]. However, if most of the light is transferred into the cladding mode and then recoupled into the fiber core, the absorption of the evanescent wave may be measured. Pu *et al.* made such an absorption sensing element from two identical LPGs that were placed 16 cm apart into a fiber loop and that transferred 99% of the light from core to cladding and back to the core mode [48]. The absorption length is larger than what is usually achieved with tapered fibers or D-shaped fibers and the insertion losses are lower than that of a microcell. Decane is transparent at the detection wavelength near 1,535 nm and has a refractive index of n = 1.412. Immersing the fiber with the LPGs into decane shifted the LPG attenuation peak to shorter wavelength by 10 nm but its spectral shape remained the same. 1-octyne (refractive index *n* = 1.417) has an absorption peak near the laser emission of 1534.3 nm and, as expected, an increase in the concentration resulted in larger losses in the fiber cavity and decreased the ring-down time. A detection limit of 0.62% 1-octyne was determined corresponding to an absorption of 150 cm^−1^.

Evanescent wave absorption is limited by the small fraction of the mode volume that has a chance of interacting with the sample. For many evanescent wave sensors shown above no more than a few percent of the light intensity can be attenuated with each roundtrip, even if the evanescent portion of the mode was completely absorbed. On the other hand, the insertion loss for many of these sensors is much lower compared to direct absorption through, for example, a gap in the fiber loop.

### 2.4. Refractive Index Measurements

**Figure 6.**
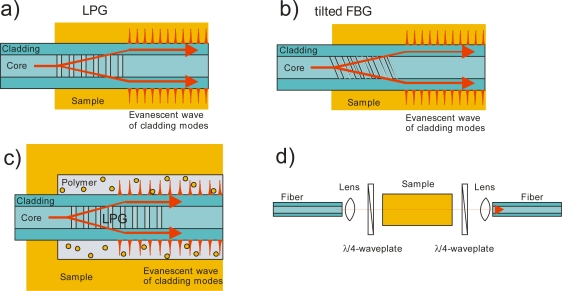
Different sensor elements for refractive index change measurements. Refractive index sensitive elements like **(a)** long-period gratings (LPG) or **(b)** tilted fiber Bragg gratings (FBGs) are often used. **(c)** By coating an LPG with a polymer the sensor element can be made more sensitive and more selective. **(d)** Circular birefringent samples have different refractive index for left or right circularly polarized light. In this sensor element the linear polarized light from the fiber is converted to circular polarized light by a quarter waveplate before being transmitted through the sample. A second quarter waveplate restores the linear polarization before coupling the light back into the cavity fiber.

Refractive index changes can also be correlated to sample concentrations and fiber-optic based refractive index sensors have a long history. Some of the most sensitive devices are based on interferometry [49] and will not be reviewed here. When a fiber cavity is used as a sensing element, the change in the refractive index of the medium surrounding the fiber can have two effects. First, the *wavelength* of the cavity mode resonances shifts when the mode’s refractive index (propagation constant) is changed. Second, the *attenuation* associated with a cavity resonance may change when refractive index sensitive components, such as LPGs, are placed into the cavity (see Figure 6). LPGs show attenuation spectra that are strongly dependent on the refractive index of the environment, and by placing them into a fiber cavity their attenuation is amplified.

The relationship between refractive index changes and concentration changes is given by the Lorentz-Lorenz equation [50]. With this equation the refractive index *n_mix_* of a mixture (volume fractions *q_1_* and *q_2_*) can be calculated when the refractive indices *n_i_* of each component is known.

(7) $$\frac{n_{\mathrm{mix}}^{2}-1}{n_{\mathrm{mix}}^{2}+2}=q_{1}\frac{n_{1}^{2}-1}{n_{1}^{2}+2}+q_{2}\frac{n_{2}^{2}-1}{n_{2}^{2}+2}$$

In very recent experiments (published in this issue of *Sensors*) Gagliardi and co-workers demonstrated refractive index measurements through both changes in the wavelength position of the cavity resonances and ring-down time (attenuation) measurements in a 2.5 m fiber loop cavity. Using a 1 mm evanescent field block they have exposed the core mode to solutions of increasing refractive index. It was found that the cavity finesse decreases and the free spectral range increases, as expected, with higher optical loss in their evanescent field block. As a consequence of the increased refractive index of the evanescent wave the mode spectrum appears red-shifted.

In an interesting variation of this theme, Song *et al*. [51] had earlier used a free-space Fabry-Pérot cavity formed between two gold-coated fiber ends to measure the refractive index and the size of living cells. A micropipette held the cell in the cavity and, again, the spectral shift in the cavity modes was measured. The shift was related to the refractive index in the cavity and by comparing the spectral shift of two buffer solutions (with different refractive indices) with and without cell in the cavity, the refractive index of the cell was determined without knowledge of the actual refractive index of the buffers. The cavity and the cell holder were incorporated into a microchip with channels for the two buffer solutions. The cavity had a length of 35.5 μm and the mirrors had a reflectivity of 80%. The refractive index and the size of Madin-Darby canine kidney cells (diameter 16–20 μm) were measured with a precision of 0.2% for the refractive index and 4.0% for their size.

There are many examples of refractive index sensors, which exploit the sensitivity of fiber gratings to the refractive index of the environment. Their optical loss can be amplified in a fiber-optic cavity. For example, Liu *et al*. [52] presented a system consisting of a linear cavity with two chirped FBGs as mirrors and a long-period grating (LPG) for refractive index change measurements (see Figure 6a). The FWHM of the FBG reflectivity was much broader than the linewidth of the laser pulses (1.50 nm *versus* 0.08 nm), and the system was relatively insensitive to thermal drifts of the FBG’s central wavelength. The LPG was chosen so that its attenuation peak was outside the reflection wavelength range of the chirped FBGs. When the surrounding refractive index changed, the attenuation peak shifted into the wavelength range of the FBGs, thereby increasing the cavity losses. The system was tested by applying index-matching liquids with refractive indices between 1.40 and 1.44 to the LPG. Liu *et al*. achieved a refractive index resolution of 9.93 × 10^−4^ at 1.410 and 4.19 × 10^−4^ at 1.440.

The LPG can also be coated with a polymer that changes its refractive index by uptake of chemicals (see Figure 6c). With customized coatings these LPG sensors can be made very sensitive, since the partition coefficient of the compound of interest can be quite high. Solid-phase microextraction then preconcentrates, say, environmental pollutants in the polymer matrix by 2 to 3 orders of magnitude. In addition, rudimentary chemical selectivity can be achieved by functionalizing the polymer coating.

We developed such a sensor with a polymer that is based on polydimethylsiloxane (PDMS) polymer which was functionalized with diphenylsiloxane units to enhance sensitivity to aromatic compounds [21]. The titanium isopropoxide crosslinker not only rigidifies the polymer but also helps adjusting the refractive index. Tuning the refractive index of the polymer coating is critical, since optimum sensitivity is achieved when the refractive index of the polymer is slightly below that of the cladding, *i.e.*, between 1.423 and 1.438 at 1,550 nm. Here, the polymer had a refractive index of 1.4237 and the thickness of the polymer coating was about 20 μm.

**Figure 7.**
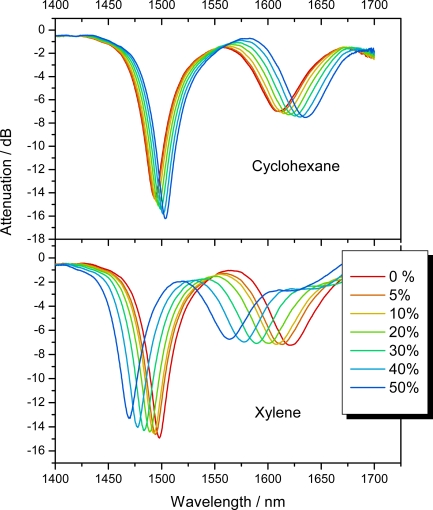
Spectra of a long-period grating (period *Λ* = 236 μm), which is coated with a polydimethylsiloxane-based polymer microextraction phase, that contained 8.5% diphenylsiloxane and 0.5% titanium cross linker. Each curve shows two attenuation features corresponding to two different low-order cladding modes. Both peaks shift as the coated LPG is exposed to 0–50% cyclohexane vapor (top panel) and xylene vapor (bottom). As the partial pressure, given here as percent of the saturation pressure, of the gas increases, the refractive index of the polymer coating decreases (increases) upon uptake of cyclohexane (xylene) causing a shift to longer (shorter) wavelengths [21].

The coated LPG was mounted in a fiber loop cavity with two fiber couplers to couple light in and out of the cavity, and phase-shift CRD was used as the detection method. Measurements of xylene and cyclohexane were performed by adding their vapors to a nitrogen flow with concentrations up to 5,400 ppm for xylene and 62,700 ppm for cyclohexane, corresponding respectively to 50% saturation pressure. Xylene has a refractive index of 1.4802 and therefore causes an increase in the refractive index of the polydimethylsiloxane (PDMS)-based coating and thus a shift of the LPG peak to shorter wavelengths. Cyclohexane has a refractive index of 1.4141, which causes the peak to shift to longer wavelengths allowing differentiation of the two substances (see Figure 7). The laser wavelength was set to the slope of the long wavelength attenuation peak so that even a small shift in peak position resulted in a large change of the loop losses. The detection limit of xylene is 320 ppm. The coating fully recovers when the vapor is removed so the same LPG and coating may be reused many times [21].

**Figure 8.**
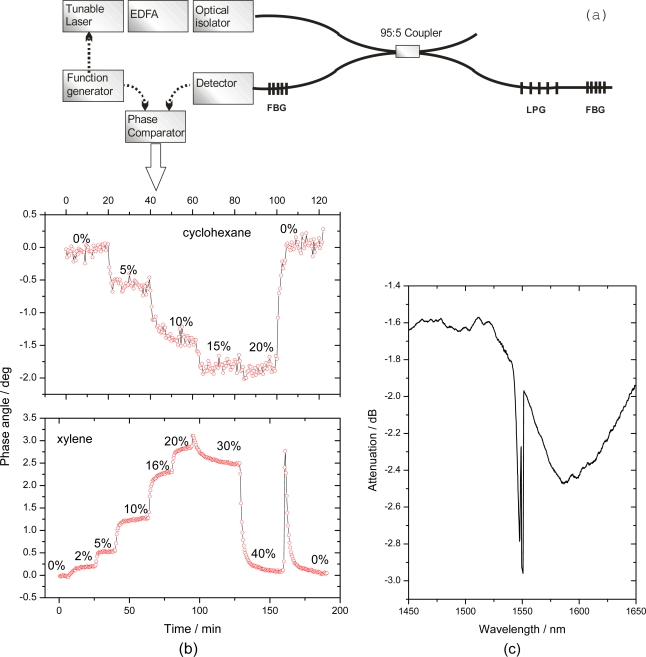
Fiber cavity ring-down for quantification of xylene and cyclohexane using a polydimethylsiloxane / polymethyloctylsiloxane coated long-period grating as a refractive index sensor. **(a)** Scheme of the experimental setup. **(b)** Response of the ring-down cavity to changes in xylene or cyclohexane concentration. As the xylene (cyclohexane) gas partitions into the polymer coating, its refractive index increases (decreases). This leads to a blue (red) shift of the LPG attenuation peak and correspondingly to a decrease (increase) of the ring-down time. At high concentrations, e.g., above 30% xylene, the LPG attenuation peak shifted so much that the FBG reflectance lies on the rising slope of the LPG attenuation peak and the ring-down time increases again. **(c)** The transmission spectrum of the cavity shows the broad LPG attenuation peak and the narrow reflection band of the two FBGs located near the mid-attenuation point.

The same chemical system was used in our group very recently on another fiber sensing platform, *i.e.*, a cavity defined by two identical narrow-band FBGs. The coated LPG was spliced into this cavity and light was introduced using a 95:5 fiber-fiber coupler (see Figure 8). A similar setup has been previously used by Bo *et al.* for temperature and strain measurements [53]. Xylene and cyclohexane were introduced at different saturation pressures between 2.5% and 50% and their concentrations were measured through the change in phase angle at 1550 nm. Laser radiation from a tunable diode laser was modulated at 300 kHz. A coating containing 35 mol% of PDMS and 65 mol% polymethyloctylsiloxane (PMOS) was used to enhance the selectivity to cyclohexane uptake and to tune the refractive index of the coating in the vicinity of 1.428, just below that of the silica cladding. The detection limit in this preliminary experiment was 1.7% saturation pressure (1900 ppm) for cyclohexane and 1.25% (140 ppm) for xylene.

A direct comparison of the fiber loop sensor and the linear fiber cavity sensor shows that the sensitivity is higher and detection limit lower for our linear fiber cavity system. This is likely due to slightly lower round trip losses and a larger light output at the detector. On the other hand, it was difficult to tune the refractive index of the coating in such a way that it would shift the attenuation peak of the coated LPG to align with the FBG cavity reflection spectrum. Calibration measurement of the etched *Λ* = 236 μm LPGs used in the both cavities show a sensitivity of 40,000 nm/RIU (RIU = refractive index unit).

A tilted FBG may be used instead of the LPG as a refractive index sensing element (see Figure 6b), as shown by Zhou *et al.* [54]. Similar to an LPG, a FBG with a large tilt angle couples narrow bandwidth radiation from the core mode to the cladding modes, but it exhibits a lower thermal sensitivity and greater insensitivity to bending. The 1 m long fiber cavity consisted of two FBGs acting as mirrors and a tilted FBG for sensing refractive index changes. As in the case of the LPG described above, the coupling between core and cladding modes caused large losses in the cavity depending on the propagated wavelength and the refractive index of the surrounding material. The attenuation peak shifted in response to the refractive index of the tilted FBG’s environment and, like the LPGs, the sensor was most sensitive in a 50 nm wavelength window near the FBG’s mid-attenuation point. As stated earlier, the cavity losses change with an increase of the environment’s refractive index, which leads to a change in ring-down time. A sensitivity of 154 μs per refractive index unit (RIU) was achieved. The system was optimized to operate in water and the largest sensitivity was near a refractive index of 1.33. By monitoring the peak position instead of the ring-down time, refractive index changes can be monitored with a sensitivity of 340 nm/RIU.

Meng *et al*. also developed and characterized a related fiber loop CRD sensor [55] for refractive index. The sensing element in their loop was a 2 cm long section of the single mode fiber that was etched from a 125 μm diameter to only 13.62 μm. The etched fiber part was immersed in dimethyl sulfoxide (DMSO) and a water-DMSO mixture. DMSO has a refractive index that is only slightly higher than that of silica and solutions of different refractive indices can be obtained by mixing water with DMSO. A model for the optical loss was developed that took optical absorption and refractive index into consideration.

Chiral liquids are a particularly interesting target for refractive index measurements. When chiral liquids are enriched in one of their two enantiomers, they become optically active, *i.e.*, the refractive indices for left and right circularly polarized light are different and the polarization plane of linearly polarized light is rotated. The circular birefringence (optical rotation) may be very small, even in pure chiral liquids. Therefore, Vollmer *et al*. [56] used fiber cavities to enlarge the effective interaction path length without increasing the sample volume. They demonstrated that the resonator modes for left- and right-circularly polarized light shift by an equal amount in wavelength but in opposite directions. The resonator is made of a single-mode fiber, of which 20 cm have been replaced by a U-bench containing collimators, quarter-wave plates and a 10 cm liquid cuvette (see Figure 6). The quarter-wave plate in front of the cuvette transforms the quasi-linear modes into left-circularly and right-circularly polarized light; the second quarter-wave plate restores the linear polarization. A tunable DFB laser at 763 nm is used to examine the two distinct resonator mode spectra associated with left and right polarized light. By inserting chiral liquids into the cuvette these two resonator mode spectra shift with respect to each other. With a wavelength shift resolution limit of 0.02 pm Vollmer *et al*. were able to quantify birefringence of only 2 × 10^−7^. Measurements of three chiral substances, limonene, pinene, and carvone, were performed. Enantiomers induce birefringence with opposite sign, and by mixing the chiral molecule with its enantiomer the amount of birefringence is adjusted. As expected, a 50:50 (racemic) mixture of enantiomers did not exhibit any birefringence, while the measured birefringence varied linearly with the amount of enantiomer added to the chiral liquid.

## 3. Amplified CRDS

Almost all sensing elements (micro-optic cells, gratings, tapers, *etc.*) introduce rather large optical losses in a fiber cavity - in addition to those caused by couplers and splices. All those losses reduce the ring-down time of the empty cavity and limit the sensitivity and detection limit of fiber based CRD measurements. In principle, this problem can be overcome by inserting a fiber amplifier to compensate for all “undesired” losses in the cavity such that, in an ideal case, very long ring-down times are obtained. Under these ultrasensitive conditions any sample interaction will significantly reduce the ring-down time, and the detection limit and sensitivity would be greatly improved.

In this ideal experiment the roundtrip gain, *G,* compensates exactly the roundtrip losses, *L_sys_*, whereas the losses due to the interaction with the sample *L_sample_* remain uncompensated. Assuming that the gain and the losses are independent of the light intensity and that their fluctuations are negligible during the ring-down event, the ring-down time is simply

(8) $$\tau =\frac{t_{\mathrm{RT}}}{-\text{ln}\;\left(L_{\mathrm{sys}}+L_{\mathrm{sample}}-G\right)}$$

A light pulse injected into such an optical cavity will decay with a time constant *τ* that depends only on the difference between the total losses and the gain, and the round-trip time *t_RT_*. As before, the ring-down time can be determined by pulsed CRDS, cw CRDS or phase-shift CRDS, but by applying an amplification much longer ring-down times can be achieved. If the gain is *larger* than the combined losses, “ring-up” occurs and the fiber cavity becomes a fiber laser.

Erbium doped fiber amplifiers (EDFAs) operate at wavelengths between 1,520 nm to 1,560 nm and are readily available due to their use in the telecommunications industry. Other less commonly used fiber amplifiers are doped with e.g., thulium to provide gain at 1460–1530 nm or 800–850 nm, with praseodymium for gain near 1.3 μm and with neodymium or ytterbium for gain around 1 μm.

Amplified cavity ring-down spectroscopy in the time domain was first demonstrated in 2001 by Culshaw and coworkers [57]. They replaced a part of the fiber loop (length of 58 m) with a 15–20 m long erbium-doped fiber amplifier, which compensates for most of the undesired loss. In their initial experiments the gain was kept below the lasing threshold and the decay of an injected laser pulses was monitored. A bandpass filter was used to select the circulating wavelength and a variable attenuator allowed for adjustment of the intracavity losses. As well, a 5 cm long open path gas cell with GRIN-lenses on both fiber ends was inserted into the loop for gas absorption measurements. A ring-down time of 2.6 μs was achieved (round trip loss: 0.48 dB); however, fluctuations in the ring-down time due to drifts in the amplifier gain were too large for trace gas sensing applications. In an improved setup the ring-down time was increased to 10 ± 2 μs (round trip loss 0.12 ± 0.02 dB) [58].

The above experiments were complicated by the fact that EDFAs are build for amplification by one to three orders of magnitude and not for gain factors of, say, 1.1999 as needed for loop losses of 20.00%. This problem can be circumvented by setting the gain above the lasing threshold, such that the fiber cavity becomes a free-running fiber laser. The EDFA is then gain-clamped to the laser threshold and ring-down measurements are possible at wavelengths different from the lasing wavelength. With such a setup Culshaw and coworkers [59] achieved ring-down times of 30 μs to 100 μs, depending on wavelength of the DFB laser. The success of the ring-down time depends upon the proximity of the lasing wavelength to the probe wavelength; the largest ring-down times are recorded when the two wavelength are near coincidence. Ring-down times up to 200 μs (0.004 dB loss per round trip) are observed under these conditions. Conversely, as the wavelengths approach one another the relaxation oscillations increase. The excited state lifetime of erbium is relatively long and these oscillations decay over a period of a few milliseconds, yet the ring-down time may still be extracted from the first half cycle [59]. Even so, the ring-down trace is partially distorted and the oscillations produce an unstable environment to take measurements. Therefore, the accuracy and reproducibility of the 200 μs ring-down time is limited. The final limiting factor of this setup was the 1 nm band pass filter bandwidth, which made it difficult to scan the wavelength of the laser as it is needed for gas spectroscopy.

Thereafter, a digital narrow band-pass filter was used to eliminate the low-frequency distortion due to relaxation oscillations and the high frequency noise [60]. This modification yields an impressive ring-down time of 10 ms. The very low loss of 0.00010 dB per roundtrip came at a cost of increased variations (up to 30%) in the ring-down time. This was attributed to drifts of the free-running laser wavelength with respect to the DFB wavelength and mode-hopping of the fiber laser. While such a setup is extremely sensitive to optical losses in the loop it is not quite adequate for the acquisition of spectra due to the necessity for the two wavelengths to be near coincidence. It was found that scanning this second “probe” laser also required changing the free-running laser wavelength and then produced relaxation oscillations. This problem may be overcome by implementing a setup using two nested loops that share the same amplifier but operate at very different wavelengths. Such a setup was described by Stewart *et al*. but was, to the best of our knowledge, not implemented [57].

**Figure 9.**
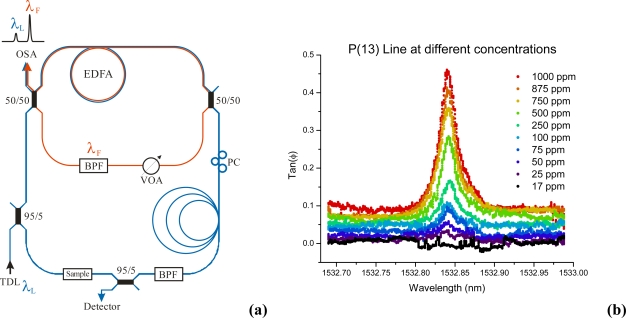
**(a)** Setup of the amplified cavity ring-down system using two fiber loops that share the same amplifier. The lasing inner loop stabilizes the gain, and the outer loop is used for the ring-down measurements. The advantage compared to the single-loop system is that the lasing wavelength and the wavelength for the spectroscopic measurement do not need to be near or at coincidence to produce ultra-long ring-down times. **(b)** Measurement of acetylene in helium; 1 bar total pressure.

Our group revisited this experiment proposed by Stewart *et al*. in 2001 [57]. Using two nested loops that shared the same EDFA, the inner loop formed a fiber laser that “clamped” the amplifier at its gain threshold, while the outer “ring-down” loop contained a sample cell and operated just below the lasing threshold (see Figure 9). The relative gain in these two loops was balanced by adjusting a variable optical attenuator. The wavelength of the inner loop was set by a narrow tunable band pass filter. The band pass filter in the outer loop was used to prevented crosstalk between the loops and was set to the wavelength of the injected laser light.

In a preliminary phase-shift CRD experiment a 6 cm gas cell, formed using two GRIN lenses, was inserted into the outer loop. By careful adjustment of the variable optical attenuator ring-down times up to 200 μs were achieved (effective roundtrip loss of 0.0163 dB). A spectrum of the P(13) line of the ν_1_+ν_3_ combination band of acetylene was obtained and a detection limit of ∼25 ppm was determined. The spectra were obtained at atmospheric pressure resulting in pressure broadened linewidth of ∼3.14 GHz. Future work will be focused on replacing the gas cell with a photonic crystal fiber to obtain a longer interaction path length and smaller sample volumes.

Ni *et al*. [61] also developed an amplified cavity ring-down system in which a digital least-mean-square filter is used to reduce ASE noise to improve the accuracy of the measurements. The fiber cavity contains two fiber couplers with 50:50 split ratio, a tunable gain EDFA, a tunable narrow band-pass filter, a optical variable attenuator and a NIST standard gas cell. The least-mean-square filter is a digital filter with self-adjusting characteristics and is used to remove ASE noise produced by the EDFA, which is the main limitation for their amplified CRD system. The pulse train of the ring-down event is filtered at once by estimating the noise. By using the least-mean-square algorithm the filter coefficients for the noise estimation improve gradually as the filter learns the characteristics of the signal. This filter reduced the error of the ring-down measurement by 9 dB at a ring-down time of 2.7 μs. With a newer system [62] the same group obtained ring-down times up to 101.2 μs corresponding to a round-trip loss of 0.218 dB. Measurements of acetylene (C_2_H_2_, at 1531.54 nm) were performed and a detection limit of 70.1 ppm and an accuracy of 15 ppm was determined. This detection limit is 8.2 times smaller than what was achieved without the filter.

Ni *et al*. used the same setup for a refractive index change sensor with a LPG [63]. The sensor is suitable for refractive index changes in the range from 1.35 to 1.43. Immersing the LPG in oils with refractive indices between 1.35 and 1.43 shifted the resonance wavelength of the LPG and therefore increased the losses of the loop. Instead of measuring the change in ring-down time, the ring-down time was kept constant by adjusting the variable attenuator. The needed attenuation then contains the information about the refractive index of the medium surrounding the LPG. The results exhibit a linear relationship between the attenuator loss and refractive indices between 1.35 and 1.43.

## 4. Conclusions

Cavity ring-down spectroscopy using either fiber loops or linear fiber cavities is well suited to amplify losses in fiber optic inline sensors. One can build sensitive, robust and inexpensive sensor systems, when these fiber optic sensors are designed to react to the optical properties associated with a chemical species of interest, such as absorption, refractive index, or birefringence. The large number of sensor head designs that are now incorporated into fiber cavities speak to the wide range of possible applications. The insensitivity to fluctuations in the light source output and detector response is a particularly attractive feature of any CRD method—including fiber-optic CRD—and has the potential to reduce operating costs and calibration for fiber sensor systems.

However, a fiber cavity may not always help increase the sensitivity of the sensor system and one must carefully consider the sensor properties. For example, the insertion loss of the sensor head, *i.e.*, its loss in the absence of analyte, may severely limit the sensitivity and detection limit. Insertion losses of over 10% are common for direct absorption cells as described in section 2.2; they are lower (<1%) for off-resonance FBGs, and depend on the design of fiber tapers, etched regions, evanescent field access blocks, and LPGs. Furthermore, almost all sensor heads have high sensitivity only when their insertion loss is also large, *i.e.*, the gain in sensitivity of the sensor is offset by the loss of sensitivity of the ring-down measurement. Consider, for example, that direct absorption sensors increase sensitivity with the width of the gap, that tapers, evanescent field blocks and etched regions are most sensitive when much of the core mode is exposed, and that high attenuation LPGs are frequently more sensitive than those with low insertion loss. Each increase in the sensor element’s sensitivity also leads to a higher insertion loss and, in the extreme, may render the use of cavity enhancement useless.

Some fiber optic components are commonly available only for single mode fibers (FBGs, LPGs) whereas others (tapers, direct absorption, field access blocks) are also available for multimode fibers. Amplified fiber loop ring-down may be a powerful (albeit expensive!) option to boost the sensitivity of single mode fiber sensors.

For sensors that may be interrogated at a single wavelength in the telecom region, the linear fiber cavity formed by two FBGs is preferred over the fiber loop, since it achieves a higher finesse and is more compact and easily coupled to the light source or detector. The fiber loop cavity is a better option, when the fiber core needs to be large (e.g., for evanescent field or direct absorption measurements), when wavelengths outside the telecom region need to be used to interrogate the sensor head, or when the light source has a broad emission spectrum. Fiber loops can also be formed from unconventional fibers, such as photonic crystal fibers or hollow core waveguides, liquid core waveguides, or infrared (ZBLAN or chalcogenide) waveguides and it appears as if a number of research groups are exploring these approaches.

Fiber-CRD measurements are still a novel tool and many research groups currently explore its applications in analytical chemistry and biochemistry. Novel applications appear with increasing frequency and it is quite possible that in 5 years the most prominent use of fiber CRD has not even been anticipated in this review.
